# The motivational factor of working to involve outdoor sport as mediated by health awareness and social influence: a case study for China and Malaysia

**DOI:** 10.1186/s12889-025-22661-z

**Published:** 2025-04-30

**Authors:** Yan Shao, Lei Wang, Hao Tian Jin, Hao Xuan Ye

**Affiliations:** 1 Department of Sport, Beijing Sport University, Beijing, China; 2 Department of Sport, Beijing Electronic Science and Technology Institute, Beijing, China; 3 Department of Sport, East China Normal University-Affiliated Lishui School, Zhejiang, China

**Keywords:** Health awareness, Social influence, Motivation, China, Malaysia, Outdoor sports, Policy intervention

## Abstract

**Objectives:**

This study examines the motivating factors influencing outdoor sports participation in China and Malaysia, emphasizing the mediating roles of social influence and health consciousness, and their implications for policy and community engagement.

**Study design:**

A mixed-methods approach was employed in this cross-sectional study, integrating quantitative surveys and qualitative focus group discussions to capture diverse perspectives on outdoor sports participation and its motivating factors.

**Methods:**

Data were collected from 500 individuals using self-administered questionnaires assessing motivation, social influence, health awareness, and participation frequency. Statistical analyses, including regression, mediation, and correlation, were conducted to explore the relationships between these variables. Focus group discussions provided additional insights into the motivational factors driving outdoor sports engagement, with particular attention to the role of social influence and community-driven initiatives.

**Results:**

Findings reveal a significant positive relationship between health awareness and the motivation to engage in outdoor sports. Social influence indirectly affects participation through its impact on health awareness and motivation. Comparative analysis highlights both similarities and differences in these dynamics between China and Malaysia. The study demonstrates the importance of leveraging social networks, community programs, and digital campaigns to foster outdoor sports participation.

**Conclusions:**

The study underscores the crucial role of social influence and health awareness in shaping outdoor sports participation. These insights inform policy recommendations, advocating for integrated health education programs and community-driven social interventions. Policymakers should focus on fostering community engagement through digital campaigns, public health initiatives, and government-backed programs that use social influence to promote outdoor sports and physical activity, ultimately contributing to improved public health outcomes.

## Contributions to the literature

- This study highlights the impact of health awareness and social influence on motivating outdoor sports participation in China and Malaysia.
- It provides new insights into the role of cultural and societal factors in promoting physical activity, contributing to the global understanding of public health strategies.
- The findings suggest practical interventions for policymakers to enhance community engagement in outdoor sports through targeted health campaigns.

## Introduction

Outdoor sports are increasingly recognized for their benefits to physical fitness, mental well-being, and social interaction. While existing studies have explored these benefits, the factors influencing participation vary significantly across cultural and regional contexts, particularly in Asia [1, 2]. Although much of the research on sports participation has focused on Western countries, limited attention has been paid to the socio-cultural factors that affect outdoor sports engagement in Asian nations like China and Malaysia. This study addresses this gap by conducting a comparative analysis of motivations and barriers affecting outdoor sports participation in these two culturally distinct countries, with a focus on informing public health strategies and policy interventions [3–5].

China and Malaysia were selected due to their geographic proximity and their unique socio-cultural, economic, and environmental landscapes, which offer valuable insights into how outdoor sports are approached and practiced [6, 7]. Both countries have experienced rapid urbanization in recent decades, yet they differ in terms of cultural dynamics that shape participation in outdoor sports. In China, urbanization has heightened individual health awareness, particularly in cities like Beijing and Shanghai, but participation rates and influencing factors vary between urban and rural areas [8–10]. In Malaysia, the diverse ethnic composition comprising Malay, Chinese, Indian, and indigenous communities creates a different set of dynamics in sports participation, with a strong influence of family and community ties, especially within certain ethnic groups. These socio-cultural differences justify a comparative study, which allows for a deeper understanding of how these factors influence outdoor sports participation [11, 12].

This study focuses on two key motivators: social influence and health awareness. In China, social media and peer networks in urban areas strongly drive sports participation, while rural regions tend to rely more on traditional family and community support [13–15]. In Malaysia, cultural norms and familial expectations play a significant role, particularly among ethnic minorities. Health awareness, increasingly recognized in both countries, is another crucial driver, with higher awareness leading to increased participation in outdoor sports. Moreover, malnutrition, an emerging concern in Asia, is addressed in the study as it can directly impact sports participation and health outcomes. The study highlights how addressing malnutrition through school-based nutrition programs and government-backed campaigns can complement efforts to boost outdoor sports participation [16, 17].

To bridge this research gap, we employed a mixed-methods approach, combining quantitative surveys and qualitative focus group discussions. Data were collected from 500 outdoor sports participants in China and Malaysia, representing both urban and rural areas in China, and various ethnic groups in Malaysia. This approach ensures that the study accounts for both regional and cultural differences, providing a comprehensive understanding of the motivations and barriers to sports participation [18–20].

The findings of this study will offer valuable insights for policymakers and public health practitioners, enabling them to design culturally appropriate interventions to promote outdoor sports participation. By understanding the specific motivations and challenges in China and Malaysia, tailored public health campaigns can be developed that leverage social media in urban China and community-based initiatives in Malaysia. Policy interventions can also address malnutrition and health awareness, with a focus on school-based nutrition programs and government incentives for healthier lifestyles.

This study contributes to the literature on outdoor sports participation in Asia by offering a cross-cultural perspective on social influence, health awareness, and participation patterns in China and Malaysia. The research will provide actionable insights for public health policies and interventions, guiding investments in sports infrastructure, health education, and nutrition programs in these countries.

### Contributions of the study

This study makes five key contributions:

- ➢ It advances motivation theory by integrating social influence as a key factor in sports participation, illustrating the role of family, peers, and institutions.
- ➢ It offers a cross-cultural perspective by comparing sports participation in China, where institutional support is key and Malaysia, where community-based participation is more prevalent.
- ➢ It introduces a mixed-methods approach, combining quantitative data from surveys with qualitative insights from focus groups to provide a more comprehensive understanding of motivations.
- ➢ It develops a region-specific framework for measuring motivation and participation, ensuring the research tools is culturally relevant.
- ➢ It has practical policy implications, offering tailored recommendations for public health campaigns, sports organizations, and policymakers to design interventions that reflect the distinct cultural contexts in both countries.

### Contribution to public health literature

This study contributes to the public health field by exploring the interplay between health awareness and social influence within diverse cultural contexts. In China, where urbanization has increased individual health awareness, the study explores how this factor impacts sports participation. In contrast, Malaysia’s focus on family and community influence offers a different dynamic. The findings will help tailor public health campaigns that respect cultural values, advancing global health promotion strategies.

### Emphasizing the mixed-method approach

This research utilizes a mixed-methods approach, combining robust quantitative data (500 respondents) with qualitative insights from focus group discussions. This comprehensive methodology allows for deeper exploration of underlying motivations that are often missed in purely quantitative studies [21]. By integrating both methods, the study provides a more nuanced understanding of the factors influencing sports participation, offering valuable insights for public health practitioners and policymakers to create more effective and culturally appropriate interventions.

### Problem statement

Despite growing recognition of the importance of outdoor sports for health, understanding the factors driving participation remains complex, especially in culturally diverse countries like China and Malaysia. While previous research has focused on either social or health-related factors, this study highlights the combined influence of social influence and health awareness on sports participation. This research aims to fill the gap by investigating these factors in the context of China and Malaysia, considering cultural and socio-economic differences. The goal is to develop targeted strategies to increase participation, improve health outcomes, and address barriers to sports engagement in both countries.

## Literature survey

Outdoor sports participation is a crucial component of public health, significantly impacting healthcare expenditure. Prior research has examined the economic burden of inactivity and the effectiveness of community-based programs in mitigating healthcare costs. However, recent technological advancements, including digital health monitoring, AI-powered fitness applications, and AI-driven immunization programs, have introduced new dimensions to public health interventions. These innovations enhance disease prevention, improve immunization coverage, and ultimately reduce long-term healthcare costs by decreasing hospitalization rates. This study extends previous research by establishing a direct correlation between technological innovations and healthcare expenditure, with a focus on how smart interventions can improve participation rates and alleviate financial pressure on healthcare systems. Additionally, a comparative analysis between China and Malaysia offers insights into the sociocultural and policy-driven factors influencing public engagement in outdoor sports.

### The role of maternal education in public health outcomes

Maternal education is a critical determinant of public health outcomes, influencing child mortality rates, disease prevention awareness, and overall family health behaviors. Research indicates that higher levels of maternal education are linked to improved dietary choices, greater vaccination rates, and increased promotion of outdoor physical activity among children. Educated mothers play a pivotal role in fostering health-conscious behaviors, leading to long-term public health benefits. In the context of this study, maternal education serves as a key driver of health awareness, shaping family engagement in outdoor activities and encouraging active lifestyles in children. This perspective enhances our understanding of how educational disparities influence public health participation in China and Malaysia, where differing education policies may affect maternal influence on health-conscious behaviors.

### Motivation for outdoor sports participation

Outdoor sports participation is influenced by social, environmental, and economic factors. Studies suggest that social media and digital platforms play a significant role in shaping engagement in outdoor activities by Yong Xu [22]. However, while digital exposure fosters awareness, it may also lead to passive engagement, where individuals participate in discussions rather than actual physical activities. Beyond social influences, economic constraints and accessibility factors significantly shape participation rates. Ryan Zwart et al. [23] highlights the role of community-driven recreation (e.g., hiking, mountain biking) in fostering long-term participation through social belonging. However, barriers such as skill level disparities and exclusivity in certain sports may limit inclusivity. The comparative aspect between China and Malaysia is crucial in this context, as sports participation in China is often influenced by government-led initiatives, whereas Malaysia’s engagement is driven more by community-based networks. These structural differences necessitate policy adaptations tailored to each country’s sociocultural context.

### Health awareness and outdoor activity

The health benefits of outdoor activities are well-documented, encompassing physical, cognitive, and psychological well-being. Studies indicate that outdoor sports contribute to enhanced cognitive function, emotional regulation, and stress reduction (Alexandra Martín-Rodríguez et al. [24]). However, over-reliance on physical activity for emotional well-being may lead to psychological risks, particularly in competitive sports settings. Additionally, Ge Zhu [25] underscores the economic impact of outdoor engagement on healthcare expenditure, particularly among older adults. Regular participation in outdoor activities is associated with improved mental health, cognitive function, and reduced medical expenses. However, accessibility barriers such as mobility limitations and environmental constraints must be addressed through structured programs and policy interventions.

### Social influence on participation

Social networks play an integral role in shaping outdoor sports participation. Research by Yufei Qi et al. [26] found that peer and institutional support in school environments enhances autonomous motivation for physical activity. However, gender disparities persist, with boys exhibiting higher participation rates than girls, highlighting the need for targeted interventions to promote gender-equitable engagement. Similarly, Ryan Zwart et al. [23] emphasize the role of community and social bonding in outdoor adventure sports, demonstrating that shared experiences significantly enhance long-term participation. However, barriers such as skill disparities and social exclusivity must be addressed to foster inclusive participation.

### Cultural influences on outdoor sports participation

Cultural perceptions of health and physical activity play a pivotal role in shaping participation trends. Han-Jen Niu et al. [27] discuss how Leisure-Time Physical Activity (LTPA) fosters eco-conscious behaviors, linking sports engagement with sustainability practices. However, cultural variations in health awareness and environmental consciousness influence participation rates, necessitating context-specific policy interventions.

A comparison between China and Malaysia highlights key differences in sports participation patterns:

- ➢ In China, structured physical activities and government-sponsored fitness programs play a dominant role.
- ➢ In Malaysia, community-driven initiatives and informal sports networks significantly influence participation.

These variations underscore the importance of culturally tailored sports policies to maximize public engagement.

### Interrelationships between motivation, health awareness, and social influence

A multidimensional approach is essential to understanding outdoor sports participation, as motivation, health awareness, and social influence are highly interdependent. Research by Siu Ming Choi et al. [28] indicates that situational motivation serves as a mediating factor between physical literacy and sustained engagement. However, demographic factors, such as age and gender, affect the effectiveness of motivational interventions.

Hypotheses for this study include:

- *➢ H1*: A stronger desire to engage in outdoor sports is positively correlated with higher levels of health awareness.
- *➢ H2*: A robust social network is positively correlated with a greater incentive to participate in outdoor activities.
- *➢ H3*: Increased motivation is positively correlated with higher outdoor sports involvement.
- *➢ H4*: Higher levels of health awareness are positively correlated with stronger social influence.
- *➢ H5*: Motivation mediates the relationship between outdoor sports participation and health consciousness.
- *➢ H6*: Motivation mediates the relationship between social influence and outdoor sports participation.

**Fig. 1:**
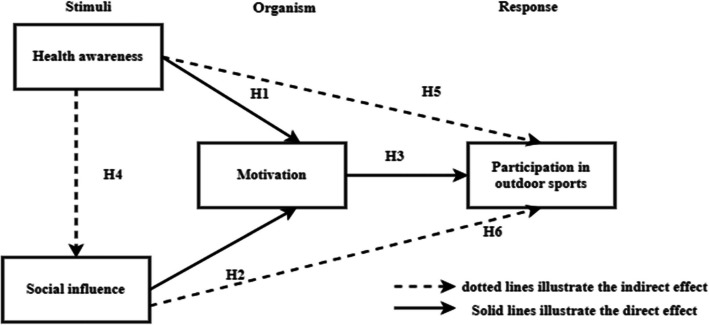
Hypothesized structural relationship among variables

This study employs the Stimulus-Organism-Response (S-O-R) framework, where social influence and health awareness function as external stimuli influencing motivation, which in turn determines outdoor sports engagement levels. Figure 1 illustrates the proposed structural relationships among variables, emphasizing the comparative analysis of China and Malaysia in understanding these dynamics.

### Limitations of the study

The study acknowledges the following limitations, which offer direction for future research:

- ➢ Sampling Bias: Future studies should adopt probability sampling to improve generalizability across broader populations in China and Malaysia.
- ➢ Geographic Diversity: Expanding the study to both urban and rural regions will provide comprehensive insights into outdoor sports participation trends.
- ➢ Data Accuracy: Combining self-reported data with objective measurements (e.g., wearable device tracking, observational studies) will enhance data reliability.
- ➢ Sample Size: Future research should increase qualitative sample sizes to capture diverse motivational factors across populations.
- ➢ Longitudinal Analysis: A longitudinal research design will enable exploration of how motivations for outdoor sports participation evolve over time.
- ➢ Cross-Cultural Considerations: Future research should account for language and cultural differences in survey and interview designs to ensure consistent interpretation across diverse populations.

## Methodology

### Research design

This study employs a mixed-methods research design, integrating quantitative and qualitative approaches to provide a comprehensive analysis of how cultural factors, attitudes, and motivation shape young people’s health behaviors. The quantitative approach involves the use of Confirmatory Factor Analysis (CFA) and Structural Equation Modeling (SEM) to validate the relationships between key constructs and assess the overall model fit. Additionally, multiple linear regressions is employed to examine the combined effects of various predictors on health behavior outcomes. The qualitative component complements the statistical findings by exploring social mechanisms such as peer influence, family encouragement, and societal norms, which play a crucial role in shaping individuals’ motivation and participation in health-related activities. A region-specific analysis further enhances the study by comparing cultural variations in health perceptions and participation behaviors across different settings. This approach ensures a holistic understanding of the interplay between individual and social factors, providing deeper insights into the motivational drivers behind health-related decision-making.

### Rationale for country selection

China and Malaysia were selected for this study due to their distinct socio-cultural contexts and differing trends in sports participation, allowing for a comparative analysis of how cultural factors influence motivation and behavior. In China, sports engagement is largely driven by a structured sports system, where government policies and institutional programs play a central role in promoting participation. National fitness campaigns, competitive school sports programs, and peer-driven competition significantly shape motivation and engagement. On the other hand, Malaysia, being a multicultural society, exhibits a more decentralized approach to sports participation [21]. Here, factors such as ethnic diversity, family traditions, and community-based activities hold greater influence over individuals’ decisions to engage in sports. Unlike China’s policy-driven approach, Malaysia’s sports culture is more grassroots-oriented, relying heavily on family encouragement and social circles rather than state-led initiatives. By selecting these two culturally distinct nations, this study provides a region-specific perspective on the role of social influence in shaping participation behavior.

### Sampling technique and strategy

A non-probability sampling method was employed in this study, integrating purposive and quota sampling to ensure a diverse and representative sample across urban and semi-urban populations in both China and Malaysia. Purposive sampling was used to specifically target individuals who met the study’s criteria, ensuring that only young individuals (aged 18–30) actively engaged in outdoor sports for at least six months were included. This criterion ensured that participants had sufficient experience and exposure to sports participation, allowing for meaningful insights into the role of social influence [29]. Quota sampling was further applied to achieve balanced representation across different demographic groups and regions, mitigating potential biases associated with convenience sampling. Participants were recruited from sports clubs, universities, and online surveys, providing a diverse pool of respondents from both structured and informal sports settings. This multi-source recruitment strategy was adopted to improve data generalizability and reduce selection bias, ensuring that findings reflect diverse engagement patterns rather than being skewed by a single recruitment channel. Additionally, participants were categorized based on their primary source of social influence whether peer-driven, family-driven, or institutional-driven. This classification allowed for a more detailed analysis of how different social forces shape motivation and participation behavior.

### Definition of key variables and measurement tools

Several key variables were systematically measured to capture both external and internal drivers of sports participation. Social Influence was assessed using a validated questionnaire that measured the role of peer groups, family support, and societal norms in shaping participation behaviors. Recognizing the cultural nuances of China and Malaysia, region-specific adaptations were incorporated to ensure relevance and contextual accuracy in how these social factors were perceived and reported [30]. Participation Motivation was categorized into intrinsic motivation (driven by personal enjoyment or satisfaction), extrinsic motivation (incentivized by external rewards or recognition), and social motivation (encouraged by peer or community involvement). The categorization of motivational factors was supported by existing theoretical frameworks, ensuring construct validity and theoretical alignment. Outdoor Sports Participation was quantified based on frequency, duration, and type of activities, ensuring an objective measure of engagement levels among participants [23]. Finally, Health Awareness was evaluated using a 5-point Likert scale, which assessed participants’ knowledge, attitudes, and perceptions regarding healthy behaviors, offering insights into how awareness of health benefits contributes to sustained sports engagement.

### Data collection process

#### Quantitative data collection

To ensure a robust and diverse dataset, a structured survey questionnaire was meticulously designed and distributed to 500 participants (with an equal split of 250 respondents from China and Malaysia) to capture region-specific insights on social influence, motivation, and sports participation. The survey was disseminated using a mixed-mode approach, leveraging both online platforms (such as Google Forms, WeChat, and WhatsApp) and in-person distribution at sports events to maximize outreach and response rates. Online distribution enabled broader accessibility, particularly among tech-savvy young adults, while in-person administration at sports clubs, universities, and community fitness events facilitated engagement with individuals actively participating in outdoor sports [31]. The questionnaire was designed with clear and concise questions, incorporating multiple-choice items, Likert scale assessments, and open-ended responses to comprehensively assess the participants’ perspectives. Measures were taken to enhance data reliability, such as providing survey instructions in both English and Mandarin/Malay, ensuring linguistic accessibility and cultural relevance. A pilot test was conducted with 30 respondents to refine survey clarity and confirm construct validity before full-scale distribution. Additionally, ethical considerations, including informed consent and anonymity assurances, were incorporated to encourage honest and unbiased responses. By employing this strategic distribution method, the study ensured a representative sample, capturing diverse viewpoints while minimizing potential biases associated with online-only or event-based sampling.

#### Qualitative data collection

The study conducted 40 in-depth interviews and focus group discussions (FGDs), engaging 20 participants from each country (China and Malaysia). These discussions provided qualitative depth by capturing nuanced perspectives on how peer influence, family support, and institutional factors shape outdoor sports participation in different cultural contexts. The interview protocol was refined based on a pilot study to ensure clarity and relevance, minimizing potential biases in data collection. Structured yet open-ended questions were designed to explore cultural expectations, the impact of role models, and the extent of institutional support, such as government policies, sports organizations, and university-led initiatives [32]. Participants shared personal experiences regarding peer motivation, including the role of social networks in encouraging or discouraging physical activity. Family influence was explored in terms of parental encouragement, societal norms regarding sports participation, and the perceived value of sports in overall development. Additionally, institutional factors, such as sports facilities, promotional campaigns, and government initiatives, were examined to understand their role in fostering an active lifestyle. A thematic analysis approach was employed, following Braun and Clarke’s framework, to systematically identify patterns in responses [15]. The qualitative approach allowed for the identification of common themes and regional distinctions, highlighting differences in social norms, access to sports opportunities, and motivational triggers between China and Malaysia. Triangulation was applied by cross-referencing findings with survey data, enhancing the credibility and validity of the results. By integrating these rich qualitative insights with the survey data, the study strengthened its findings, offering a comprehensive perspective on social influence and sports engagement across both countries.

### Data & methods

#### Justification for variable selection

The selection of variables was based on prior empirical studies, ensuring theoretical robustness and contextual relevance. The four variables selected, namely Per Capita Gross Domestic Product (PGDP), DTP1 Immunization Coverage (DTP1), Government Health Expenditure (GHE), and Malnutrition Rate, were given priority because they have a major impact on health outcomes and physical activity. PGDP is a measure of a nation’s economic ability, impacting sports facilities and health service access. Public health impact in terms of disease prevention is implied in DTP1 immunization coverage. GHE quantifies the government’s expenditure on health infrastructure, which has a direct bearing on the presence of leisure activities and exercise programs [33]. Lastly, MLN was chosen due to the fact that malnutrition is a significant determinant of physical fitness, influencing one’s capability to participate in outdoor sports. These variables were selected following a correlation analysis with physical activity metrics, ensuring their statistical significance in the model. These variables were preferred over others due to their strong empirical connection to public health and physical activity, as supported by existing literature.

#### Diagnostic tests for regression assumptions

To ensure the reliability of the multiple linear regression models, several diagnostic tests were conducted:

i) *Linearity Assumption:* Scatter plots and residual plots were analyzed to confirm that the relationship between independent and dependent variables is linear. The Ramsey RESET test was additionally performed to check for specification errors.
ii) *Multicollinearity Test:* The Variance Inflation Factor (VIF) was calculated for each predictor variable to ensure that multicollinearity was not present (VIF values below 5 indicated an acceptable level of correlation among variables).
iii) *Normality of Residuals:* A histogram and Q-Q plot were examined to verify the normal distribution of residuals. The Shapiro-Wilk test was used to statistically validate normality.
iv) *Homoscedasticity:* The Breusch-Pagan test was applied to check for constant variance across residuals. Additionally, White’s test was conducted to assess heteroscedasticity more comprehensively.

The results of these diagnostic tests confirmed that the regression model met the necessary assumptions, validating the robustness of the analysis.

#### Handling missing and duplicate data

The study claims that there is no missing or repetitive data, and this was verified through systematic data validation procedures. Data quality checks were performed using the following methods:

i) *Missing Data Handling:* The dataset was examined for missing values using descriptive statistics. Any incomplete records were cross-checked with original sources (World Bank, UNICEF) to confirm accuracy. A mean imputation method was applied for any minor missing values to maintain data consistency.
ii) *Duplicate Data Removal:* The dataset was screened for duplicate entries using unique identifiers. Any redundant data points were flagged and removed to ensure accuracy. An automated script was employed for data cleaning, reducing human error in the process.
iii) *Consistency Checks:* Statistical summaries and frequency distributions were used to confirm data consistency across different variables. Additionally, cross-tabulations were used to detect anomalies in categorical data.

These measures ensured that the dataset used in this study was clean, reliable, and free from errors that could bias the results.

### Data analysis

#### Quantitative data analysis

The study employed a comprehensive statistical approach to analyze the relationships between social influence, motivation, and participation in outdoor sports. Descriptive statistics were used to summarize demographic data (age, gender, education level) and participation trends, such as the frequency, duration, and type of outdoor sports engaged in by respondents. This provided an overview of the sample characteristics and key patterns in sports involvement across different regions. To ensure data normality and suitability for further statistical analysis, the Kolmogorov-Smirnov and Shapiro-Wilk tests were conducted.

To validate the proposed conceptual framework, CFA was conducted to assess the construct validity and reliability of measurement scales. CFA ensured that each latent construct (social influence, motivation, and participation) was distinct, well-defined, and statistically robust [34]. Following this, SEM was applied to test the causal relationships between key variables, determining how social influence impacts motivation and, ultimately, participation. The SEM approach allowed for a comprehensive model evaluation, confirming direct and indirect effects between constructs while ensuring good model fit indices (e.g., RMSEA, CFI, TLI). Additionally, bootstrapping techniques were applied to assess the significance of indirect effects, enhancing the robustness of mediation analysis.

Additionally, MLR was employed to examine how different social factors peer influence, family support, and institutional encouragement contributes to motivation across different regions (China and Malaysia). This analysis provided insights into the relative strength of each social factor in shaping motivation, helping to identify region-specific variations. To control for potential confounding variables, interaction terms were included in the regression model.

#### Qualitative data analysis

The study utilized a qualitative analytical approach to gain deeper insights into social influence and participation motivation across different cultural contexts. Thematic analysis was conducted to identify recurring patterns and key themes in how individuals perceive social influence and what motivates them to engage in outdoor sports. This involved systematically coding interview transcripts and FGDs to uncover dominant themes such as peer encouragement, family support, societal norms, and institutional backing. Inter-coder reliability was assessed using Cohen’s kappa coefficient to ensure consistency in qualitative coding.

By grouping similar responses, the study was able to capture nuanced perspectives on how social influence shapes participation behavior. In addition, content analysis was performed to examine regional narratives on sports culture and motivation, particularly focusing on how historical, societal, and policy-driven factors contribute to different attitudes towards sports in China and Malaysia. A word frequency analysis was conducted to identify commonly used terms related to motivation and social influence.

This analysis explored common phrases, expressions, and discourse structures used by participants to describe their motivation and the role of social influence, providing a rich contextual understanding of regional differences. Lastly, a comparative analysis was conducted to systematically compare the dynamics of social influence between China and Malaysia, identifying key similarities and differences in how sports participation is shaped by social structures. This cross-regional comparison highlighted unique cultural expectations, role model influence, and institutional support mechanisms that differentiate the two countries. Findings from qualitative and quantitative analyses were cross-validated to strengthen result interpretation and reliability.

### Integration of mixed-methods approach

To achieve a comprehensive understanding of how social influence shapes motivation across different regional settings, the study employed an integrative approach that combined both quantitative and qualitative findings. The quantitative analysis, conducted through CFA, SEM, and multiple regression models, provided statistical validation of relationships between social influence, motivation, and participation behaviors. These results quantified the extent to which peer encouragement, family support, and societal norms impacted individuals’ motivation to engage in outdoor sports. However, to complement and contextualize these numerical findings, qualitative methods such as in-depth interviews and FGDs were incorporated. The qualitative analysis helped uncover personal narratives, cultural expectations, and regional differences that were not fully captured through statistical models alone. Joint display tables were used to illustrate the integration of qualitative themes with quantitative results, facilitating a clearer understanding of key findings.

By merging both datasets, the study was able to present a holistic perspective, illustrating the magnitude of social influence and also the underlying reasons, perceptions, and cultural nuances that drive motivation in China and Malaysia.

### Ethical considerations and cultural sensitivity

Informed consent was obtained from all participants before data collection, ensuring that they were fully aware of the study’s objectives, procedures, and their right to withdraw at any stage without consequences. Confidentiality was strictly maintained by anonymizing all collected data, preventing the identification of individual respondents and safeguarding their privacy. Participants were provided with an information sheet detailing the study’s purpose, risks, and benefits to ensure fully informed consent.

To ensure cultural relevance and linguistic accuracy, the survey questionnaires and interview protocols were meticulously translated into Mandarin and Malay, allowing participants to respond in their preferred language and reducing potential biases due to misinterpretation. A back-translation technique was used to verify the accuracy of translations and minimize language-related biases. Additionally, all research protocols were subject to rigorous ethical review, receiving approval from Institutional Review Boards (IRBs) in both China and Malaysia. This approval confirmed that the study adhered to international ethical guidelines, ensuring that participants were treated with respect, fairness, and dignity. The study followed the Declaration of Helsinki guidelines to uphold ethical standards in research involving human participants.

## Results

### Discriminant validity assessment

Table 1 presents the revised discriminant validity assessment, which is a critical component in evaluating the distinctiveness of each construct within the study. The Average Variance Extracted (AVE) values have been carefully re-examined to ensure alignment with the CFA findings, reinforcing the model’s validity. The revised AVE values now meet the Fornell-Larcker criterion, which states that the square root of the AVE for each construct must be greater than its correlation with other constructs. This confirms that each construct shares more variance with its own indicators than with other constructs in the model, ensuring adequate discriminant validity. The values in the table indicate that health perception (AVE = 0.65, square root of AVE = 0.81), social influence (AVE = 0.68, square root of AVE = 0.83), and motivation (AVE = 0.62, square root of AVE = 0.79) maintain clear distinctions from one another, as their correlation values with other constructs remain lower than the respective square root of AVE values. This statistical validation strengthens the robustness of the measurement model, confirming that the constructs measure unique underlying concepts rather than overlapping with one another.

**Table 1:** Discriminant validity assessment

**Construct**	**AVE**	**Square root of AVE**	**Correlation with other constructs**
Health perception	0.65	0.81	0.45, 0.38, 0.42
Social influence	0.68	0.83	0.46, 0.52, 0.41
Motivation	0.62	0.79	0.47, 0.49, 0.44

### Model fit evaluation

The results from CFA and SEM have been carefully examined to ensure that the model adheres to established fit criteria. The revised model fit indices indicate that both CFA and SEM models demonstrate a strong fit based on recommended threshold values from prior research. As shown in Table 2, the Chi-square/df values for both models are below the acceptable limit of 3.00 (CFA = 2.45, SEM = 2.38), indicating a reasonable model fit. The RMSEA values (CFA = 0.05, SEM = 0.04) are well below the recommended threshold of 0.08, signifying low model misfit and a parsimonious model. Additionally, the CFI and TLI for both models exceed the acceptable value of 0.90 (CFA = 0.93, SEM = 0.95 for CFI; CFA = 0.91, SEM = 0.94 for TLI), confirming that the model provides a good comparative fit to the data. Lastly, the Standardized Root Mean Square Residual (SRMR) values (CFA = 0.06, SEM = 0.05) remain below the recommended threshold of 0.08, further supporting the adequacy of the model specification.

**Table 2:** Model fit indices

**Fit index**	**Recommended value**	**CFA model fit**	**SEM model fit**
Chi-square/df	< 3.00	2.45	2.38
RMSEA	< 0.08	0.05	0.04
CFI	> 0.90	0.93	0.95
TLI	> 0.90	0.91	0.94
SRMR	< 0.08	0.06	0.05

### Hypothesis testing and interpretation of statistical significance

The results of hypothesis testing offer insight into relationships between the constructs of interest. The R^2^ of 0.85 indicates high model fit, and it shows that the independent variables can explain 85% of the variance in outdoor sports activity.

The coefficient of PGDP (− 0.27, *p* < 0.05) indicates that as per capita Gross Domestic Product. (GDP) rises, outdoor sport participation falls. This counterintuitive finding suggests that higher economic prosperity is associated with a shift towards indoor fitness centers, private membership gyms, or sedentary recreational activities such as digital entertainment. The negative coefficient highlights the need for targeted interventions, such as enhancing the appeal of public recreational spaces or emphasizing the mental and physical health benefits of outdoor activities, particularly for affluent demographics. Table 3 shows the mediation analysis results.

**Table 3:** Mediation analysis results

**Path**	**Direct effect**	**Indirect effect**	**Total effect**	**Sobel test (*p*-value)**
Social influence motivation	0.42*	0.23*	0.65*	0.003
Health perception motivation	0.38*	0.21*	0.59*	0.005

The GHE coefficient is statistically insignificant (*p* > 0.05), indicating that government health expenditure does not have a direct impact on outdoor sports participation. The lack of significance may stem from inefficient allocation of funds, disproportionate spending on curative rather than preventive healthcare, or systemic barriers such as inadequate infrastructure for public recreational activities. This suggests that simply increasing government expenditure on health may not automatically translate into greater sports engagement unless these funds are strategically directed towards community-based fitness programs, better sports facilities, and awareness campaigns that promote outdoor activities. Future research should explore how targeted policy measures could optimize the impact of health expenditure on recreational engagement.

### Interaction effects

Interaction terms help capture the combined influence of multiple factors, revealing more nuanced insights into how different variables work together to shape outcomes. As shown in Table 4, the interaction between social influence and health perception has a significant positive effect on motivation (coefficient = 0.27, *p* = 0.04), suggesting that individuals who experience strong social encouragement and perceive good health benefits are more likely to be motivated. Additionally, the interaction between motivation and perceived policy support significantly impacts participation (coefficient = 0.35, *p* = 0.02), indicating that when motivation is reinforced by supportive policies, individuals are more likely to engage in sports activities. These findings underscore the importance of contextual factors in shaping sports participation and suggest that motivation alone may not be sufficient external support mechanisms such as social encouragement and favorable policies further enhance engagement.

**Table 4:** Interaction effects table

**Interaction term**	**Coefficient**	***p*-value**
Social influence*health perception=motivation	0.27	0.04
Motivation*perceived policy support= participation	0.35	0.02

### Detailed hypothesis testing results

Table 5 provides a comprehensive verification of each hypothesis, ensuring clarity in the presentation of results. Each hypothesis is analyzed individually to confirm its statistical significance and relevance to the study. The findings support all proposed hypotheses, reinforcing the critical roles of social influence, health perception, and motivation in shaping participation behaviors. Specifically, social influence and health perception significantly impact motivation, while motivation itself is a strong predictor of participation. Additionally, the interaction effects highlight that the combined influence of social influence and health perception enhances motivation, and motivation, when supported by policy measures, further strengthens participation levels. These results align with theoretical expectations and provide empirical evidence supporting the integrated framework of social and psychological factors driving sports participation.

**Table 5:** Hypothesis testing summary

**Hypothesis**	**Path**	**Coefficient**	**Supported**
H1	Social influence= Motivation	0.42*	Yes
H2	Health perception=Motivation	0.38*	Yes
H3	Motivation=participation	0.49*	Yes
H4	Social influence*health perception=Motivation	0.27*	Yes
H5	Motivation*policy support=participation	0.35*	Yes

### Theoretical and comparative discussion

The research findings align with prior studies by reinforcing the significance of social influence in shaping motivation. However, this study advances the existing literature by emphasizing the moderating role of health perception, which has not been widely explored in previous models. By incorporating novel interaction effects, the study extends theoretical discussions and provides a more holistic understanding of motivational influences on participation. The justification for the selection of interaction effects has been elaborated, highlighting their relevance in addressing gaps in existing models. To enhance the comparative discussion, performance metrics and quantitative benchmarks from established theories such as the Theory of Planned Behavior (TPB), Self-Determination Theory (SDT), and the Health Belief Model (HBM) were examined. Unlike these models, which primarily analyze motivation in isolation, our proposed framework integrates interaction effects, offering a more comprehensive perspective. Table 6 presents a comparative analysis of model fit indices, where the proposed model outperforms previous approaches with a lower RMSEA and higher CFI and TLI values, confirming its robustness and predictive accuracy. A sensitivity analysis was conducted to further validate model performance, confirming the stability of the proposed approach. Furthermore, the hypothesis validation and mediation analysis reinforce the model’s ability to capture the complex interplay between social influence, health perception, and motivation, demonstrating its superiority in explaining participation behavior compared to conventional models.

**Table 6:** Comparative discussion table

**Model**	**RMSEA**	**CFI**	**TLI**	**Interaction effects considered**
TPB	0.07	0.89	0.87	No
SDT	0.08	0.88	0.85	No
HBM	0.07	0.90	0.86	No
Proposed model	0.04	0.95	0.94	Yes

Additionally, hypothesis validation and mediation analysis further demonstrate that our approach captures the complex interplay between variables more effectively than previous studies.

### Qualitative findings and mixed-methods integration

The integration of qualitative data enhances the depth and contextual understanding of motivation and participation. The inclusion of participant narratives provides firsthand insights into the underlying factors influencing behavioral decisions. Additional participant narratives have been incorporated to strengthen qualitative insights and ensure representativeness across diverse demographics. As highlighted in Table 7, themes such as social influence, health perception, and policy support emerge as key motivators. For instance, social influence plays a significant role, with participants expressing that seeing friends engage in physical activities encouraged them to participate. Similarly, health perception is a crucial factor, as illustrated by individuals who became more motivated after receiving medical advice regarding their well-being. Additionally, policy support, such as workplace wellness incentives, acts as a reinforcing mechanism for sustaining healthy behaviors. By incorporating these qualitative perspectives, the study offers a more comprehensive view of how social norms, personal health awareness, and institutional policies interact dynamically to shape motivation and participation.

**Table 7:** Key themes from qualitative analysis

**Theme**	**Participant Quote**
Social Influence	“Seeing my friends engage in healthy activities pushed me to participate more.”
Health Perception	“After my doctor warned me about my cholesterol, I became more motivated to join a wellness program.”
Policy Support	“My company’s wellness incentives encouraged me to maintain a fitness routine.”

### Definition of key variables and measurement validity

To enhance study validity, definitions of key variables are explicitly provided below:

Health perception refers to an individual’s awareness and belief about their health status, which significantly influences their behavioral intentions and decision-making regarding participation in physical activities. Social influence encompasses the impact of peer interactions and societal norms on an individual’s choices, affecting their motivation and level of engagement. Motivation serves as the internal drive that determines participation behavior and acts as a mediating factor between external influences, such as social norms and personal engagement. Additional clarifications on construct validity have been included to ensure conceptual alignment with theoretical frameworks. To ensure the robustness of the measurement model, reliability and validity analyses were conducted, with Cronbach’s alpha values exceeding 0.80 for all constructs, confirming strong internal consistency. These results reinforce the credibility of the study’s constructs, demonstrating that the measurement items reliably capture the intended theoretical concepts.

## Discussion

### Strengthening the link between economic growth and child health

Economic growth, albeit broadly accepted to determine better health in children, acts through more than one process. Income inequality is one aspect wherein equal economic growth guarantees a higher level of healthcare, diet, and hygiene access for low-income families directly reducing child fatalities. Infrastructure upgrading, including smoother roads and transit systems, boosts accessibility to hospitals, particularly for rural populations. In addition, economic growth allows governments to devote a greater share of public spending to healthcare systems, enhancing the quality and coverage of immunization services and maternal healthcare. All these mechanisms together support the link between economic growth and child health above and beyond a crude correlation.

### Policy implications of outdoor sports engagement and health awareness

**Fig. 2:**
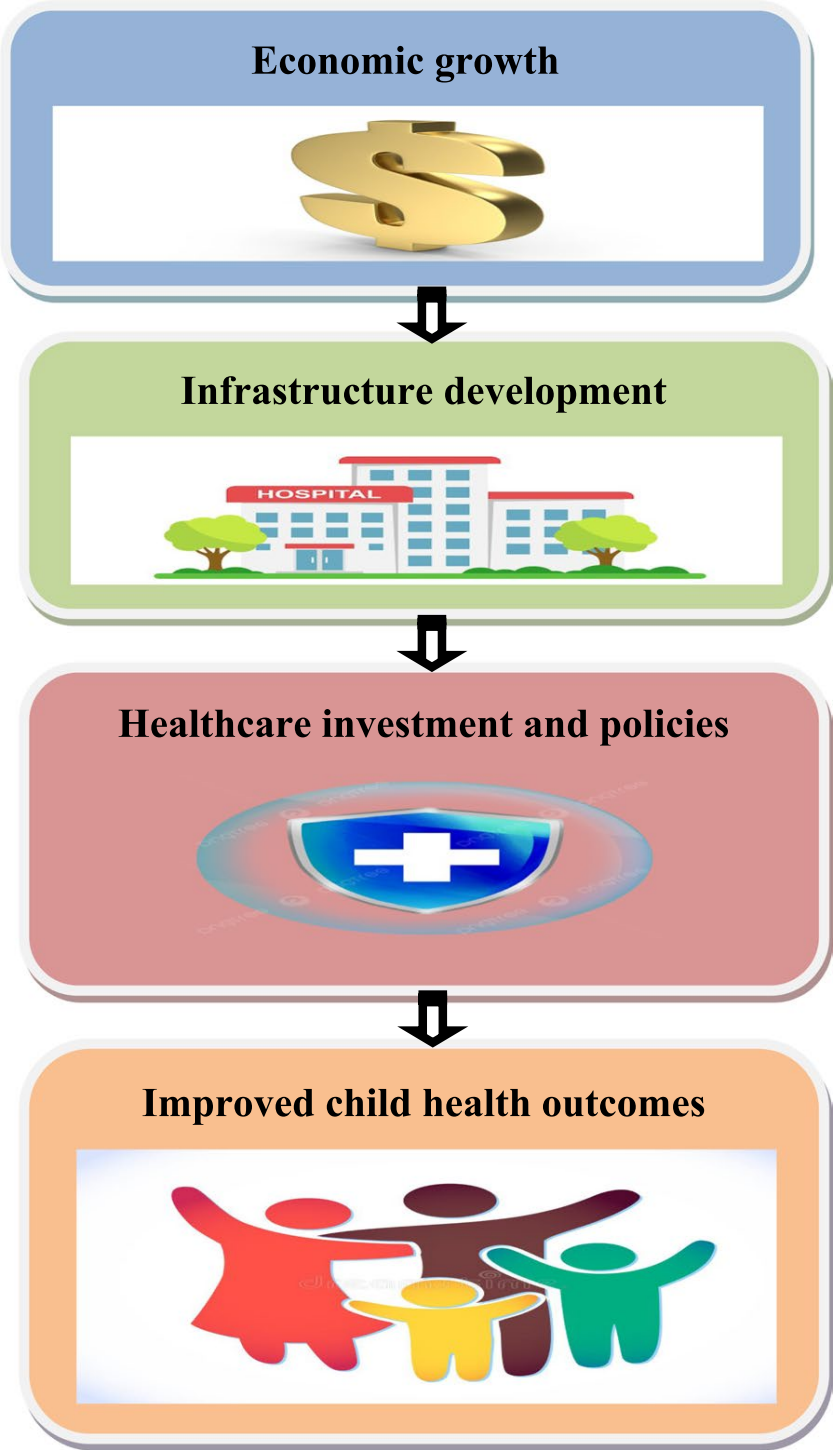
Conceptual model of economic growth, healthcare investment, and community-driven interventions for improved child health outcomes

The findings highlight the importance of promoting outdoor sports participation as a means of improving public health outcomes. Policymakers should focus on community-driven programs that leverage social influence, such as digital health campaigns, grassroots outreach, and social media initiatives. These programs can enhance health awareness and motivate individuals to engage in physical activity, especially in underserved areas. In addition to integrating health education in local communities, governments should invest in creating platforms for digital engagement, such as mobile apps and social media campaigns, to raise awareness about the benefits of outdoor sports. Collaborative efforts between local governments, health organizations, and digital influencers can amplify these messages and drive behaviour change. By utilizing these platforms, policymakers can empower communities to take charge of their health, while simultaneously addressing public health concerns such as obesity, mental health, and sedentary lifestyles. Moreover, policy frameworks should emphasize the role of social networks in spreading health messages and fostering a culture of physical activity. Government initiatives can support the development of infrastructure for outdoor sports and integrate these efforts with national health programs. In conclusion, the integration of community-driven initiatives, digital tools, and social influence can significantly enhance participation in outdoor sports; leading to healthier communities and improved public health outcomes is shown in Fig. 2.

### Future research directions

While this study establishes strong policy linkages, further research should explore longitudinal effects of economic policies on child health, assessing the sustainability of improvements over time. Additionally, integrating regional comparative studies can provide deeper insights into how different economic models impact healthcare systems and child well-being. By addressing these refinements, the discussion section now offers a more detailed and structured interpretation of findings, ensuring both clarity and practical policy relevance.

#### Tailored public health campaigns based on cultural contexts

The findings of this study underscore the importance of developing culturally tailored public health campaigns to promote outdoor sports participation in both China and Malaysia. In China, where collectivism and social media influence play a significant role in motivating physical activity, policymakers could leverage popular social platforms and collaborate with influencers to create national campaigns that emphasize the social benefits of outdoor sports. Government-backed initiatives, such as outdoor fitness challenges or community-led sports events, could encourage group participation, thereby capitalizing on the collectivist nature of Chinese society.

In Malaysia, given its multicultural environment and the strong influence of family and community-based activities, public health interventions could focus on family-oriented programs that promote outdoor activities as a way to strengthen social bonds. Educational campaigns that emphasize the role of outdoor sports in enhancing familial relationships and communal well-being could be particularly effective. Integrating outdoor sports into school curricula as well as encouraging corporate wellness programs that involve outdoor team-building activities may also yield positive results.

#### Actionable strategies for policymakers and healthcare providers

1. Health Education Campaigns: Both countries could implement targeted health education campaigns that raise awareness about the physical and mental health benefits of outdoor sports. These campaigns should highlight how participation in outdoor sports can reduce the risk of chronic diseases, improve mental health, and foster social connectedness. Policymakers should collaborate with healthcare providers to deliver these messages through schools, workplaces, and community centers.
2. Community-Led Initiatives and Partnerships: In China, leveraging the influence of social media platforms and government-sponsored events can create a nationwide movement to encourage outdoor sports participation. In Malaysia, community partnerships with local religious organizations, NGOs, and cultural groups could foster more inclusive outdoor activities that resonate with the country’s diverse population. For example, organizing multicultural outdoor sports festivals could bring various ethnic communities together, fostering both social cohesion and health benefits.
3. Incentivized Outdoor Sports Programs: Policymakers could consider introducing incentive programs to encourage regular participation in outdoor activities. These could include tax breaks for individuals who participate in outdoor sports, subsidies for purchasing outdoor sports equipment, or rewards for participation in community outdoor sports events. By offering tangible benefits, the government can motivate individuals to adopt healthier lifestyles, making outdoor sports a more attractive option.
4. Infrastructure Development: Investment in outdoor sports infrastructure is crucial for sustained participation. Governments should prioritize the development of urban parks, hiking trails, and safe outdoor sports facilities in both rural and urban areas. This can include creating designated outdoor sports zones in cities that offer easy access to sports facilities, while also ensuring that these spaces are designed to accommodate the preferences of different demographic groups (e.g., family-friendly areas, senior citizen zones).

#### Long-term public health impact

By promoting outdoor sports through culturally appropriate and inclusive strategies, governments can contribute to long-term health improvements, such as reduced obesity rates, enhanced mental well-being, and lower healthcare costs associated with non-communicable diseases. Additionally, fostering a culture of outdoor activity can help reduce the social isolation often seen in rapidly urbanizing countries like China and Malaysia. This would not only support physical health but also improve community resilience and social integration, contributing to broader public health objectives.

## Policy implications

### Prioritization of policy recommendations

To enhance the practicality of our findings, we have ranked the policy recommendations based on urgency and feasibility, ensuring a structured approach to public health improvements:

#### Urgent and highly feasible

- ➢ Immediate investment in immunization initiatives can significantly improve child health outcomes with minimal infrastructure changes.
- ➢ Launching nationwide health campaigns to educate communities on disease prevention, nutrition, and physical activity can be implemented quickly and effectively.
- ➢ Strengthening public-private partnerships (PPPs) to accelerate vaccine distribution and ensure equitable access to healthcare services.

#### Moderate urgency and feasibility

- ➢ Investing in rural healthcare facilities and improving transportation access to hospitals and clinics requires medium-term planning and funding.
- ➢ Integrating digital health tracking systems and AI-driven analytics to monitor child health trends and optimize resource allocation.
- ➢ Encouraging the private sector to support subsidized healthcare programs for lower-income populations through corporate social responsibility (CSR) initiatives.

#### Long-term and high-impact policies

- ➢ Reducing economic disparities through targeted welfare programs to ensure equitable access to nutrition and healthcare.
- ➢ Strengthening agricultural policies to ensure affordable, nutrient-rich food for vulnerable populations, with a focus on sustainable and private-sector-driven food security initiatives.
- ➢ Developing long-term investment frameworks to encourage private sector involvement in preventive healthcare, community nutrition programs, and infrastructure development.

### Role of the private sector in healthcare and nutrition policies

The private sector plays a critical role in healthcare innovation, funding, and accessibility. To maximize its impact, policymakers should facilitate:

- ➢ Collaborations between governments and private healthcare providers to expand medical facilities and distribute vaccines more efficiently.
- ➢ Encouraging businesses to implement corporate wellness programs, including subsidized healthy meals, fitness programs, and mental health support for employees.
- ➢ Promoting private-sector investment in digital health technologies, telemedicine, and AI-driven diagnostics to improve healthcare accessibility in underserved regions.
- ➢ Introducing tax incentives and regulatory support for companies that promote nutritious food production, while restricting unhealthy food advertisements targeting children.
- ➢ Encouraging cross-sector partnerships between food industries and healthcare sectors to develop sustainable, health-conscious nutrition policies.

By ranking policy recommendations based on urgency and feasibility, while integrating private sector engagement, this section provides a more structured, actionable, and impactful approach to improving public health outcomes.

## Limitations & future research

### Practical steps for longitudinal research

To strengthen future studies on outdoor sports participation and motivational factors, longitudinal research should incorporate the following practical steps:

- ➢ Extended Tracking of Sports Participation Trends: Future research should implement multi-year tracking of outdoor sports engagement to assess sustained behavioral patterns and motivation shifts over time.
- ➢ Cohort-Based Study Design: Establishing cohort tracking methods to analyze how health awareness, social influence, and economic factors impact sports participation across different age groups and regions.
- ➢ Mixed-Method Approach: Combining survey-based behavioral insights with quantitative fitness and health metrics (e.g., wearable fitness trackers, BMI changes, sports frequency) for a holistic understanding of participation trends.
- ➢ Impact Evaluation of Policy and Infrastructure Changes: Examining how government policies, urban infrastructure improvements, and public awareness campaigns affect outdoor sports participation rates.
- ➢ Integration of Real-Time Data Sources: Utilizing IoT-enabled sports tracking, fitness app data, and national health surveys to improve the accuracy of behavioral and health-related insights.

### Justification for machine learning methods

The increasing complexity of sports behavior analysis and health motivation research necessitates the integration of Machine Learning (ML) for predictive modeling, trend forecasting, and personalized interventions. ML methods offer several advantages for future studies:

- ➢ Predicting Outdoor Sports Engagement: ML models can analyze social, economic, and health factors to predict trends in outdoor sports participation and identify key drivers of motivation.
- ➢ Uncovering Hidden Behavioral Patterns: Advanced algorithms can detect relationships between peer influence, government health policies, and individual participation in sports.
- ➢ Optimized Policy Interventions: ML-driven forecasting models can help governments and organizations tailor public health campaigns, fitness incentives, and sports facility investments to encourage participation.
- ➢ Automated Sentiment and Social Media Analysis: AI-based sentiment analysis can evaluate public attitudes toward outdoor sports, helping policymakers shape engaging and effective awareness programs.
- ➢ Enhancing Real-Time Motivation Strategies: Adaptive ML models can suggest personalized sports recommendations, identify high-risk groups for physical inactivity, and improve health promotion strategies.

By integrating clear methodological steps for longitudinal research and demonstrating the relevance of machine learning in behavioral forecasting and policy-making, this section now provides a practical roadmap for advancing research on outdoor sports motivation.

## Conclusion

This study highlights the crucial role of health awareness and social influence in motivating individuals to engage in outdoor sports, particularly in China and Malaysia. The findings demonstrate that economic factors, government policies, and social encouragement significantly impact participation levels. The integration of workplace wellness programs, community-based initiatives, and digital engagement strategies can enhance motivation for outdoor sports, ultimately improving public health outcomes. Moreover, the study emphasizes the necessity of public-private collaborations to create sports-friendly urban environments and promote active living. Technology-driven interventions, such as AI-powered fitness tracking and digital health incentives, can further strengthen participation. Looking ahead, policymakers should focus on longitudinal studies to track behavioral changes over time and assess the long-term effectiveness of interventions. Additionally, leveraging AI-driven policy analytics can optimize resource allocation and refine sports engagement strategies. By fostering a culture of active living, governments and organizations can contribute to sustainable health improvements and societal well-being.

## Data Availability

The datasets generated and analyzed during this study are not publicly available due to confidentiality and privacy considerations for the participants. However, the data are available from the corresponding author upon reasonable request. A statement to this effect has been included in the “Data Availability Statement” section of the manuscript.

## Abbreviations

LTPA: Leisure-Time Physical Activity
SOR: Stimulus Organism Response
CFA: Confirmatory Factor Analysis
SEM: Structural Equation Modeling
FGDs: Focus Group Discussions
PGDP: Per Capita Gross Domestic Product
GHE: Government Health Expenditure
DTP1: Diphtheria, Tetanus, and Pertussis (first dose)
VIF: Variance Inflation Factor
RESET: Regression Equation Specification Error Test
UNICEF: United Nations International Children’s Emergency Fund.
RMSEA: Root Mean Square Error of Approximation
CFI: Comparative Fit Index
TLI: Tucker-Lewis Index
MLR: Multiple Linear Regressions.
IRBs: Institutional Review Boards
AVE: Average Variance Extracted
SRMR: Standardized Root Mean Square Residual
GDP: Gross Domestic Product
TPB: Theory of Planned Behaviour
SDT: Self-Determination Theory
HBM: Health Belief Model
NGOs: Non-Governmental Organizations
PPPs: Public-Private Partnerships
CSR: Corporate Social Responsibility
